# Comprehensive proteomic analysis reveals omega-3 fatty acids to counteract endotoxin-stimulated metabolic dysregulation in porcine enterocytes

**DOI:** 10.1038/s41598-023-48018-3

**Published:** 2023-12-07

**Authors:** Tamil Selvi Sundaram, Maria Filippa Addis, Carlotta Giromini, Raffaella Rebucci, Salvatore Pisanu, Daniela Pagnozzi, Antonella Baldi

**Affiliations:** 1 Department of Veterinary Medicine and Animal Sciences, University of Milan, Via Dell’Università 6, 26900 Lodi, Italy; 2 University of Veterinary Medicine and Pharmacy in Košice, Komenského 68/73, 04181 Košice, Slovakia; 3 Porto Conte Ricerche S.R.L, S.P. 55 Porto Conte/Capo Caccia, Loc. Tramariglio 15, 07041 Alghero, Italy

**Keywords:** Protein-protein interaction networks, Protein-protein interaction networks

## Abstract

Omega-3 polyunsaturated fatty acids (n-3 PUFA), such as the eicosapentaenoic acid (EPA) and docosahexaenoic acid (DHA), are reported to beneficially affect the intestinal immunity. The biological pathways modulated by n-3 PUFA during an infection, at the level of intestinal epithelial barrier remain elusive. To address this gap, we investigated the proteomic changes induced by n-3 PUFA in porcine enterocyte cell line (IPEC-J2), in the presence and absence of lipopolysaccharide (LPS) stress conditions using shotgun proteomics analysis integrated with RNA-sequencing technology. A total of 33, 85, and 88 differentially abundant proteins (DAPs) were identified in cells exposed to n-3 PUFA (DHA:EPA), LPS, and n-3 PUFA treatment followed by LPS stimulation, respectively. Functional annotation and pathway analysis of DAPs revealed the modulation of central carbon metabolism, including the glycolysis/gluconeogenesis, pentose phosphate pathway, and oxidative phosphorylation processes. Specifically, LPS caused metabolic dysregulation in enterocytes, which was abated upon prior treatment with n-3 PUFA. Besides, n-3 PUFA supplementation facilitated enterocyte development and lipid homeostasis. Altogether, this work for the first time comprehensively described the biological pathways regulated by n-3 PUFA in enterocytes, particularly during endotoxin-stimulated metabolic dysregulation. Additionally, this study may provide nutritional biomarkers in monitoring the intestinal health of human and animals on n-3 PUFA-based diets.

## Introduction

Chronic gut inflammation, characterized by conditions like intestinal barrier damage, leads to pathogenesis of various gastrointestinal disorders including the inflammatory bowel diseases (IBD)^1^. Over the decades, dietary omega-3 polyunsaturated fatty acids (PUFAs) such as the eicosapentaenoic acid (EPA; C20:5n-3) and docosahexaenoic acid (DHA; C22:6n-3) have gained considerable attention in controlling chronic inflammation and associated diseases^2,3^. Generally, these essential fatty acids are enriched in the phospholipid cell membranes, that serves as a precursor to specialized lipid-based signalling molecules called eicosanoids^4,5^. The eicosanoids regulate the entire inflammatory process from initiation, progression, to resolution^6–9^. Therefore, the family of eicosanoid incorporates molecules of both pro- and anti-inflammatory actions. Various mechanisms have been proposed for the anti-inflammatory effects of n-3 PUFA. These include the production of eicosanoid molecules with 100-fold weak pro-inflammatory action than n-6 PUFAs, and generation of pro-resolving eicosanoids such as the resolvins, protectins, and maresins^8–12^. In support of these reports, numerous in vitro and in vivo studies as summarized earlier in our review, have shown n-3 PUFA to potentially reduce the cardinal signs of IBD including the expression of pro-inflammatory cytokines, chemokines, adhesion molecules, inflammatory cell infiltration, and tissue damage in the gut^1^. Also, in these studies, n-3 PUFAs improved the expression of tight junction proteins such as occludin and claudins, thereby reducing the intestinal permeability to harmful substances and pathogens. Furthermore, n-3 PUFAs were shown to support the production of mucus, which acts as a protective barrier alongside epithelia in maintaining gut homeostasis^1,13^. In these early in vitro studies, anti-inflammatory effects of n-3 PUFA was largely demonstrated using cancer enterocyte models^1,14,15^. Cancer cell models pose certain limitations such as aberrant cell surface sugars, glycoproteins, and metabolic pathways^16,17^.

Recently, we demonstrated the cytoprotective properties of n-3 PUFA in an in vitro inflammatory model of small intestinal barrier, established using the porcine IPEC-J2 cells^18^. IPEC-J2 is a non-cancer and non-transformed cell line, isolated from the jejunum epithelium of unsuckled piglets. Jejunum is the primary site of fatty acid absorption, which makes IPEC-J2 a suitable model for n-3 PUFA assessment. Moreover, previous gene expression study showed IPEC-J2 to retain most of the biomarkers intrinsic to native epithelial structure and response, especially towards host–pathogen interactions^19^. In this cell model, we assessed the effects of n-3 PUFA in the presence and absence of inflammation and oxidative damage^18^. The pathophysiological events of these stress conditions were stimulated in the IPEC-J2 cells using different biological and chemical stressors such as the lipopolysaccharides, hydrogen peroxide, and dextran sodium sulphate. The outcome of our study showed n-3 PUFA to promote enterocyte proliferation under normal conditions^18^. Upon challenge with different stress factors, n-3 PUFA prevented cellular damage by increasing the mitochondrial activity and cell membrane integrity, while inhibiting enterocyte apoptosis^18^. These observations show, by targeting both gut inflammation and intestinal barrier damage, n-3 PUFAs and their metabolites offer a comprehensive approach to managing gastrointestinal disorders. Up to date, there is no report on transcriptome or proteome of enterocytes exposed to n-3 PUFA in normal and under its state of infection. Therefore, the biological pathways corresponding to n-3 PUFA’s mechanism of action in intestinal barrier are poorly described. Presently, we established a shotgun proteomics workflow to comprehensively map the changes in proteomic profile of IPEC-J2 cells exposed to n-3 PUFA (DHA:EPA), LPS or n-3 PUFA pre-treatment followed by LPS stimulation. In addition, RNA-sequencing (RNA-seq) was employed to compare the gene expression with differential protein abundance data. Subsequently, the biological pathways in which the differential proteins were involved was accessed using different open-source proteomics databases.

## Results

### Experimental design

**Figure 1:**
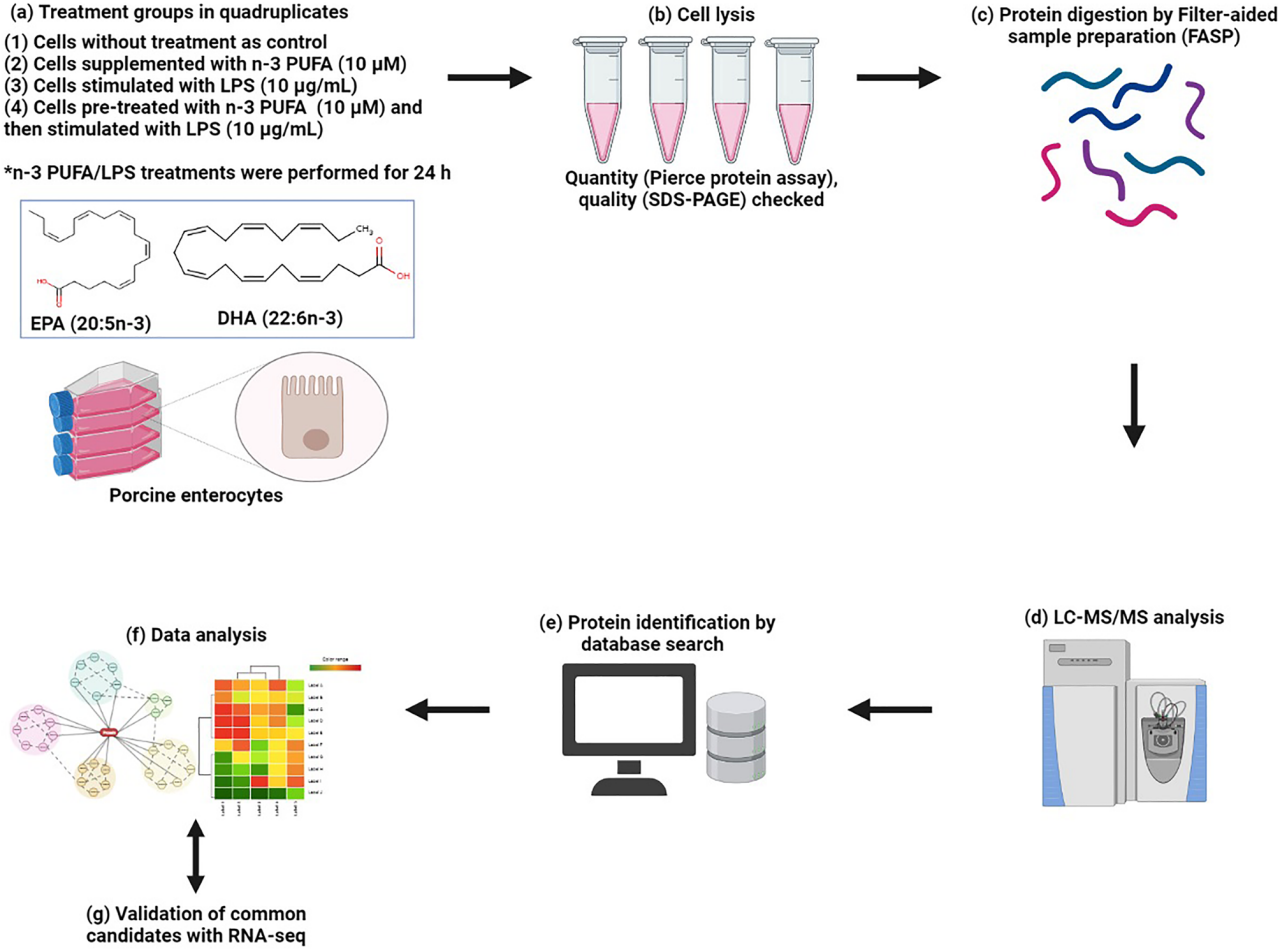
Schematic representation of the experimental workflow. (**a**) Treatments were performed on enterocyte monolayer cultures established using the porcine IPEC-J2 cell line. (**b**–**c**) Cells were lysed, proteins extracted, and enzymatically digested. (**d**) Peptide mixture was separated and analysed by LC–MS/MS. (**e**–**f**) Proteins were identified and analysed using Proteome Discoverer and open-source databases. (**g**) Differential proteins were validated using RNA-seq analysis. Figure created with BioRender.com.

To comprehensively map the proteomic profiles of porcine IPEC-J2 cells exposed to n-3 PUFA (DHA:EPA, 1:2, 10 µM, 24 h), LPS (10 µg/mL, 24 h), or 24 h n-3 PUFA pre-treatment followed by 24 h LPS stimulation (n-3 PUFA + LPS), the shotgun proteomic analysis was employed. Cells contained ethanol carrier was included as a control, and all the experimental group was composed of four biological replicates. The concentrations and incubation time of test compounds were chosen based on our previous assessment of its dose–response, cell viability, cytotoxicity, and apoptosis effects^18^. The differential analysis was performed by comparing each treatment group against the control; in particular, (i) n-3 PUFA versus control, (ii) LPS versus control, and (iii) n-3 PUFA + LPS versus control. The proteomics results were validated by comparing them against the RNA-seq analysis performed under identical experimental conditions. Figure 1 illustrates the experimental design used in this study.

### Differentially abundant proteins

**Figure 2:**
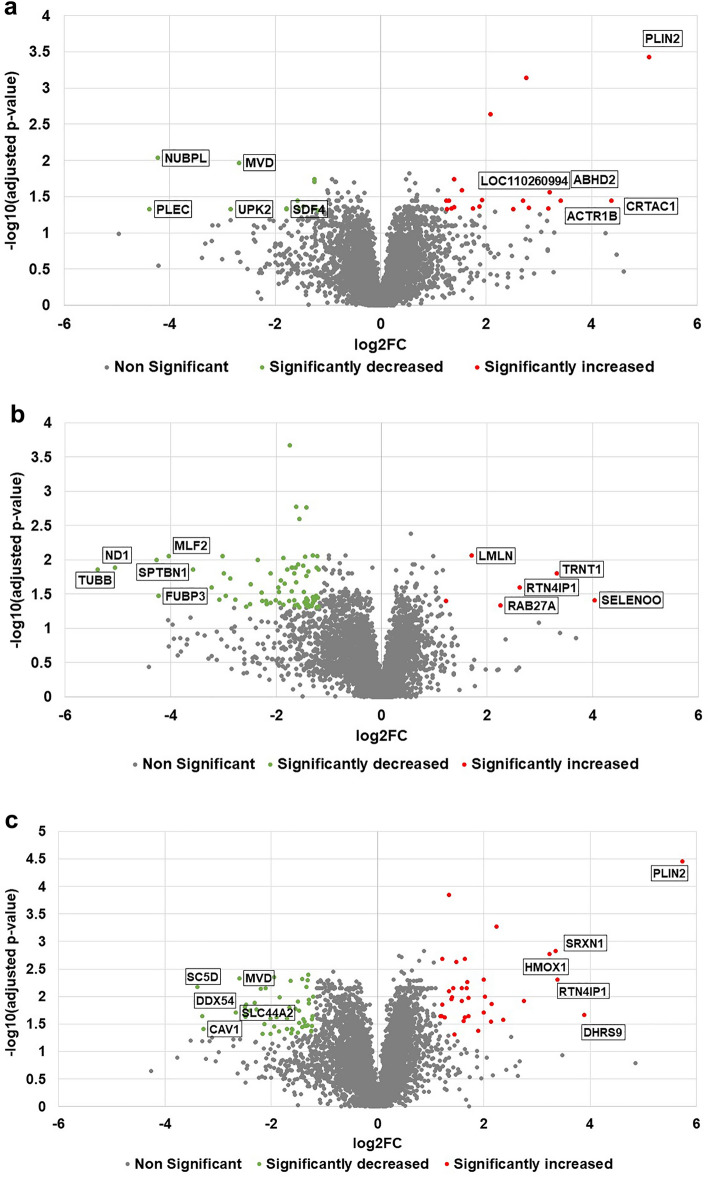
Volcano plot displaying the differential proteins identified in the porcine IPEC-J2 cells exposed to (**a**) n-3 PUFA, (**b**) LPS, or (**c**) n-3 PUFA + LPS treatments. Red dots represent significantly enhanced proteins, and green dots represent significantly decreased proteins, while the grey dots represent non-significant proteins after applying log_2_FC ≥ 1.2 or ≤ − 1.2 and adjusted *p*-value ≤ 0.05 cut-offs. The top five protein candidates with the highest and lowest log_2_FC values are indicated in the plots.

**Figure 3:**
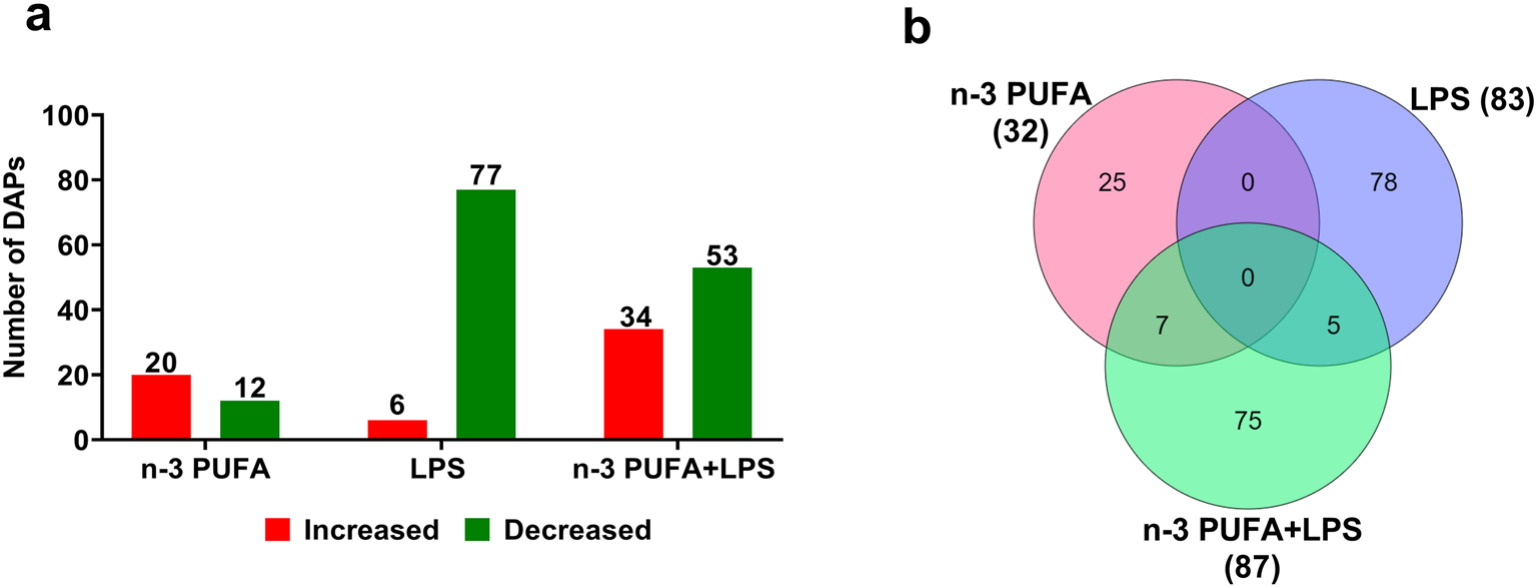
(**a**) Bar graph shows the number of increased (red) and decreased (green) differentially abundant proteins (DAPs), and (**b**) Venn diagram shows the number of shared and unique DAPs identified in the IPEC-J2 cells exposed to n-3 PUFA, LPS, or n-3 PUFA + LPS treatments (log_2_FC ≥ 1.2 or ≤  − 1.2; adjusted *p*-value ≤ 0.05).

In average, 5481 ± 35.6 proteins were identified in each treatment group upon comparison with the control. After applying log_2_FC ≥ 1.2 or ≤ -1.2 and adjusted *p*-value ≤ 0.05 cut-offs, 32 (20 increased and 12 decreased), 83 (6 increased and 77 decreased), and 87 (34 increased and 53 decreased) proteins were found to be statistically significant in n-3 PUFA (Figs. 2a–3a and Supplementary file Dataset 1.0), LPS (Figs. 2b–3a, Supplementary file Dataset 1.1), and n-3 PUFA + LPS (Figs. 2c–3a, Supplementary file Dataset 1.2) treatment groups, respectively. As shown in the Venn diagram (Fig. 3b), n-3 PUFA uniquely regulated 25 proteins in the IPEC-J2 cells and shared 7 proteins with n-3 PUFA + LPS treatment group. Similarly, LPS uniquely regulated 78 proteins and shared 5 proteins with n-3 PUFA + LPS treatment group. Moreover, n-3 PUFA + LPS treatment group uniquely regulated 75 proteins in the IPEC-J2 cells. However, none of the differential proteins were in common between the three analyses.

### Gene ontology and pathway enrichment analysis

**Figure 4:**
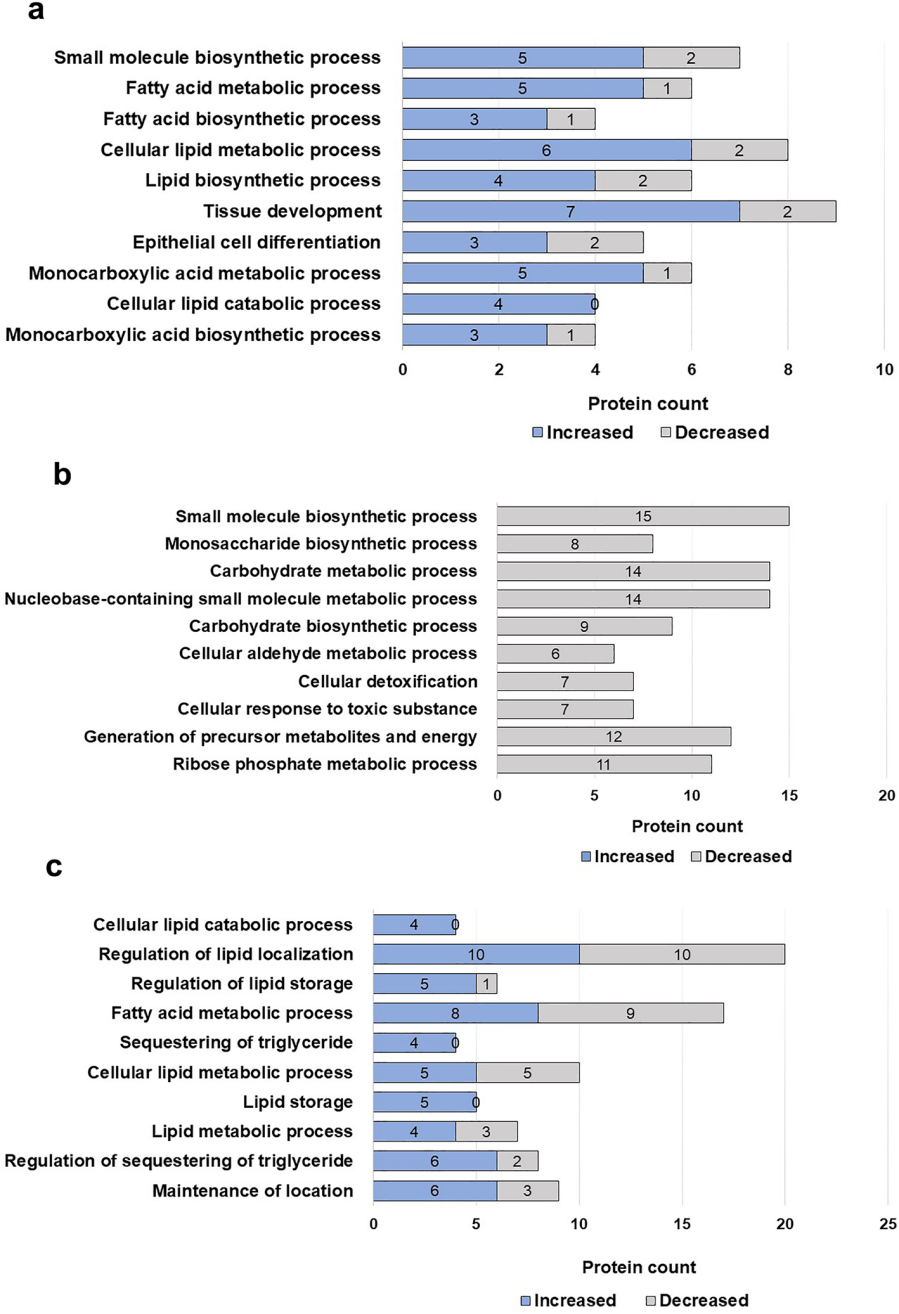
Gene Ontology (GO) annotations of the differentially abundant proteins (DAPs) obtained in the IPEC-J2 cells exposed to (**a**) n-3 PUFA, (**b**) LPS, or (**c**) n-3 PUFA + LPS treatments. The plots display the number of DAPs in the top ten GO biological processes (FDR < 0.05). The significantly enhanced DAPs are represented in blue, and the decreased in grey.

In the GO analysis, n-3 PUFA-regulated DAPs were mapped to a total of 556 biological processes, of which 12 were significant (FDR < 0.05). These processes were mainly related to fatty acid metabolism, epithelial differentiation, and development (Fig. 4a, Supplementary file Dataset 2.0). The DAPs regulated by LPS were mapped to 346 biological processes, of which 110 were significant (FDR < 0.05). Interestingly, the abundance of all the DAPs annotated in LPS treatment group was profoundly decreased. These DAPs were mainly involved in small molecular and carbohydrate metabolic processes (Fig. 4b, Supplementary file Dataset 2.1). On the other hand, 441 biological processes were mapped by the DAPs identified in n-3 PUFA pre-treated cells after stimulation with LPS. Notably, 73 of these biological processes, including triglyceride sequestration, lipid storage, and metabolism were significant (FDR < 0.05) (Fig. 4c, Supplementary file Dataset 2.2). Unlike LPS, the abundance of most of the DAPs identified in n-3 PUFA and n-3 PUFA + LPS treatment groups were increased.

### Protein–protein interaction network

**Figure 5:**
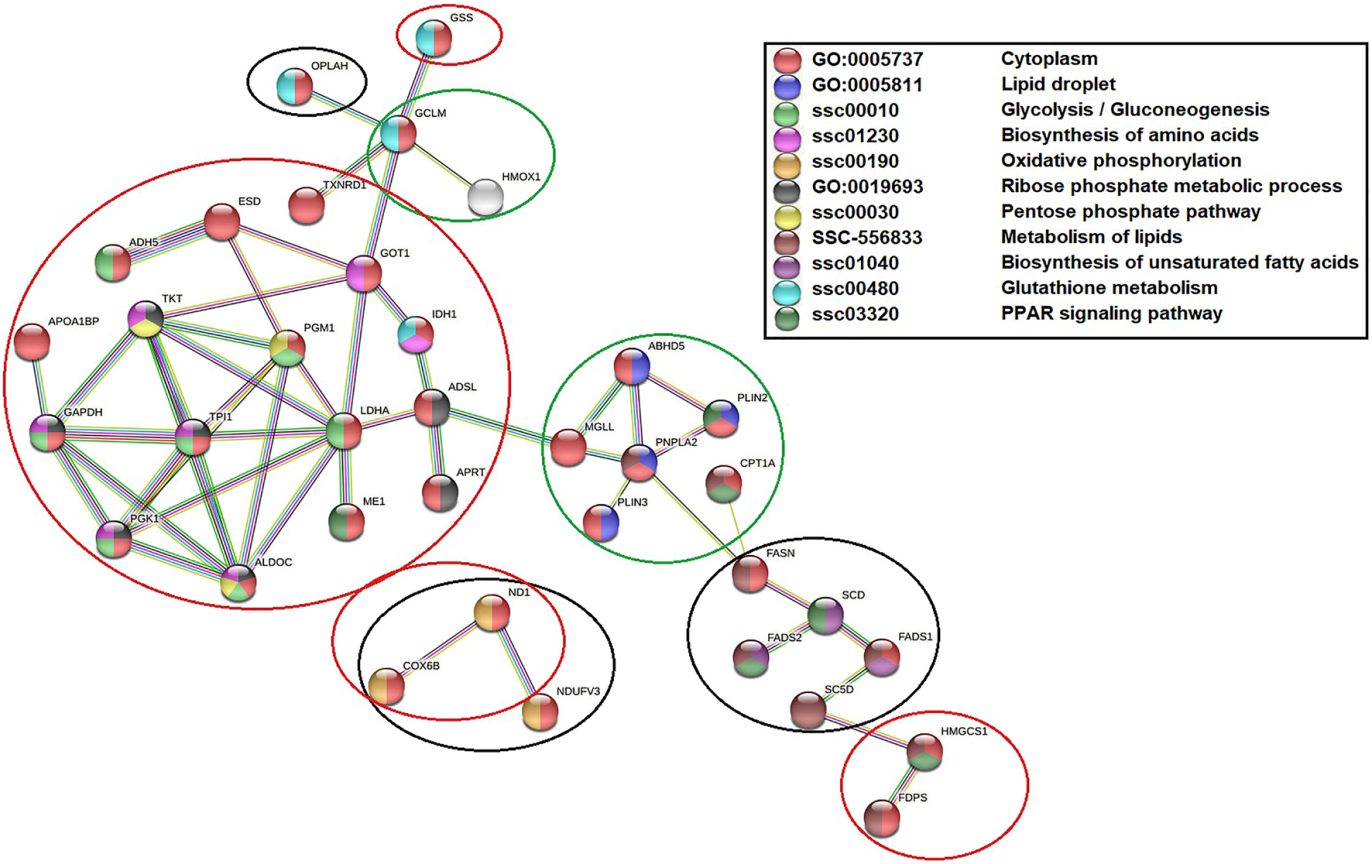
STRING protein–protein interaction network. The network showed DAPs whose abundance (*p* < 0.05) decreased in the IPEC-J2 cells post-stimulation with LPS were encircled in red. The highly abundant proteins (*p* < 0.05) in n-3 PUFA + LPS treated cells were encircled in green, while the low abundant proteins (*p* < 0.05) were encircled in black. In the network, proteins are represented as nodes and the connecting links as edges. Node colour denotes the biological pathways to which the proteins were mapped. Edge colour indicates the evidence used in predicting the protein–protein associations. Light blue edge: curated databases; pink edge: experimentally determined; green edge: gene neighborhood; red edge: gene fusions; blue edge: gene co-occurrence; yellow edge: text mining; black edge: co-expression; purple edge: protein homology.

As LPS treatment primarily induced changes in the cellular metabolism, the DAPs mapped in the Reactome pathway, ‘Metabolism (R-HSA-1430728)’ (*p* < 0.05) pathway was selected to further elucidate the role of n-3 PUFA pre-treatment in regulating the abundance and interaction of metabolic proteins following LPS stimulation. Although this pathway was not significantly implicated (*p* > 0.05) in cells treated with only n-3 PUFA, it is still reported for comparison with other treatment groups. Accordingly, in all the treatments together, 61 DAPs (12 increased and 49 decreased) were identified (Table 1, Supplementary file Dataset 3.0–3.2). Among these DAPs, 36 were significantly (p < 0.05) mapped in the STRING PPI networks for the set confidence (0.7) (Fig. 5). The proteins are represented as nodes and the connecting links as edges in a network. The disconnected nodes were removed from the network obtained.

As shown in Fig. 5 (red nodes), most of the DAPs mapped in the PPI network were associated with the cytoplasm. Interestingly, the abundance of PLIN2 (Perilipin 2; log_2_FC = 5.75), PLIN3 (Perilipin 3; log_2_FC = 1.71), ABHD5 (1-acylglycerol-3-phosphate O-acyltransferase; log_2_FC = 2.14) and PNPLA2 (Patatin Like Phospholipase Domain Containing 2; log_2_FC = 1.6) proteins that corresponds to lipid droplets in cytoplasm was significantly enhanced in n-3 PUFA + LPS treatment group (Fig. 5, violet nodes). None of these proteins were regulated by LPS but PLIN2 (log_2_FC = 5.09) alone was enhanced by n-3 PUFA treatment. Like ABHD5, the abundance of ABHD2 was also enhanced by n-3 PUFA irrespective of LPS stimulation (Supplementary file Dataset 1.0 and 1.2), but it did not map in the Reactome metabolic pathway chosen for PPI analysis. Further, 6 cytoplasmic proteins whose abundance decreased by LPS including ALDOC (Fructose-bisphosphate aldolase C; log_2_FC = − 1.4), TPI1 (Triosephosphate Isomerase 1; log_2_FC = − 1.7), GAPDH (Glyceraldehyde-3-phosphate dehydrogenase; log_2_FC = − 1.43), PGK1 (Phosphoglycerate kinase 1; log_2_FC = -1.56), ADH5 (Alcohol Dehydrogenase Class-III), and LDHA (Lactate Dehydrogenase A; log_2_FC = − 1.22) were mapped in the ‘Glycolysis/Gluconeogenesis (ssc00010)’ pathway (Fig. 5, green nodes). In the same network, PGM1 (Phosphoglucomutase 1; log_2_FC = − 1.47) abundance was reduced only in the n-3 PUFA treatment group. The proteins such as TKT (Transketolase; log_2_FC = − 1.7), GOT1 (Aspartate aminotransferase; log_2_FC = -3.22), and IDH1 (Isocitrate dehydrogenase [NADP]; log_2_FC = − 1.43) in addition to those found in Glycolysis/Gluconeogenesis pathway were mapped in the ‘Biosynthesis of amino acids (ssc01230)’ network (Fig. 5, magenta nodes). Notably, n-3 PUFA pre-treatment prevented the LPS from inhibiting all the proteins in these two pathways.

In the network of ‘Oxidative phosphorylation (ssc00190)’ pathway, LPS decreased the abundance of ND1 (NADH-ubiquinone oxidoreductase chain 1), and COX6B (Cytochrome c oxidase subunit) proteins (Fig. 5, dark yellow nodes). As reported earlier in this manuscript, n-3 PUFA pre-treatment prevented the LPS from suppressing ND1 (log_2_FC of − 5.06 to − 2.31), but moderately supported the inhibition on COX6B (log_2_FC of − 1.1 to − 1.42). In the same network, NDUFV3 (NADH ubiquinone oxidoreductase 9 kDa subunit; log_2_FC = − 1.42) abundance was only reduced in the n-3 PUFA + LPS treatment group. Besides, the abundance of 7 proteins mapped in the ‘Ribose phosphate metabolic process (GO:0,019,693)’ network as the ALDOC, PGK1, GAPDH, TPI1, TKT, ADSL (Adenylosuccinate lyase; log_2_FC = − 1.95), and APRT (Adenine phosphoribosyltransferase; log_2_FC = − 1.41) were significantly reduced by LPS treatment (Fig. 5, grey nodes). Among these proteins, ALDOC, TKT, and PGM1 were also mapped in the ‘Pentose phosphate pathway (ssc00030)’ (PPP) (Fig. 5, yellow nodes). Notably, n-3 PUFA counteracted the LPS-stimulated inhibition of these proteins.

Antioxidant enzymes such as the GSS (Glutathione synthetase), GCLM (Glutamate-cysteine ligase modifier subunit), and OPLAH (5-oxoprolinase, ATP hydrolysing) were associated with the ‘Glutathione metabolism (ssc00480)’ network (Fig. 5, blue nodes). In this network, LPS uniquely reduced the protein abundance of GSS (log_2_FC = − 2.49) and IDH1. This effect was inhibited upon prior treatment with n-3 PUFA. In the n-3 PUFA + LPS treatment group, the abundance of GCLM (log_2_FC = 2) was uniquely enhanced, while the OPLAH (log_2_FC = − 2.49) was decreased.

Furthermore, String analysis highlighted a total of 8 proteins mapped in the ‘Metabolism of lipids (SSC-556833)’ pathway. These proteins included the PNPLA2 (Patatin Like Phospholipase Domain Containing 2), CPT1A (Carnitine O-palmitoyltransferase 1A), FASN (3-hydroxyacyl-[acyl-carrier-protein] dehydratase (Fragment)), FADS(Fatty Acid Desaturase)1, FADS2, FADS4 (also known as SC5D), FADS5 (also known as SCD), HMGCS1 (3-hydroxy-3-methylglutaryl coenzyme A synthase), and FDPS (Farnesyl pyrophosphate synthase). The proteins such as FADS1, FADS2, and FADS5 were also mapped to the ‘Biosynthesis of unsaturated fatty acids (ssc01040) pathway (Fig. 5, purple nodes). Likewise, CPT1A, FADS2, FADS5, HMGCS1 in addition to ME1 (Malic Enzyme 1; log_2_FC = − 2.77), and PLIN2 were mapped in the ‘PPAR signaling pathway (ssc03320)’ network (Fig. 5, dark green nodes). Among these proteins, n-3 PUFA abolished the LPS-stimulated inhibition on HMGCS1 (log_2_FC = − 1.46), ME1 (log_2_FC = − 2.77), and FDPS (log_2_FC = − 1.66). Moreover, the abundance of PNPLA2 (log_2_FC = 1.62) and CPT1A (log_2_FC = 1.48) were uniquely enhanced in the n-3 PUFA + LPS treatment group, while the FADS1 (log_2_FC = − 2.13), FADS2 (log_2_FC = − 2.4), FADS4 (log_2_FC = − 1.9), and FADS5 (log_2_FC = -2.4) were decreased. In the same treatment group, the abundance of CPT1C (log_2_FC = 1.35) enzyme that exhibits a similar function to CPT1A was also enhanced; however, it was not mapped in the network. Notably, treatment with n-3 PUFA alone did not regulate any of these proteins, but it uniquely reduced the abundance of FASN (log_2_FC = 1.62).

**Table 1:** DAPs involved in the metabolism of IPEC-J2 cells exposed to n-3 PUFA, LPS, or n-3 PUFA + LPS treatments (log_2_FC ≥ 1.2 or ≤ -1.2 with adjusted *p*-value ≤ 0.05).

Protein ID	Protein description	Gene name	n-3 PUFA versus Ctrl (log_2_FC)	LPS versus Ctrl (log_2_FC)	n-3 PUFA + LPS versus Ctrl (log_2_FC)
Q079S8	Nucleotide binding protein like	NUBPL	− 4.23	0	0
A0A4X1SLV1	Diphosphomevalonate decarboxylase	MVD	− 2.69	0	− 2.6
P00503	Phosphoglucomutase 1	PGM1	− 1.47	0	0
F1RQM4	3-hydroxyacyl-[acyl-carrier-protein] dehydratase	FASN	− 1.28	0	0
A0A4X1T7V5	L-lactate dehydrogenase	LDHA	− 1	− 1.22	0
P50578	Sterol-C5-desaturase	SC5D	0	0	− 3.39
A0A4X1SH01	Caveolin 1	CAV1	0	0	− 3.28
F6QAM9	Choline transporter-like protein 2 isoform X1	SLC44A2	0	0	− 2.67
A0A5G2QES7	5-oxoprolinase, ATP hydrolysing	OPLAH	0	0	− 2.49
A0A4X1SLI2	Peptidyl-prolyl cis–trans isomerase	PPIL3	0	0	− 2.47
A0A288CFT0	Cytochrome b5 heme-binding domain-containing protein	FADS2	0	0	− 2.4
A8U4R4	NADH-ubiquinone oxidoreductase chain 1	ND1	0	− 5.06	− 2.31
A0A481C945	Fatty acid desaturase 1a	FADS1	0	0	− 2.13
A0A4X1T629	Thioredoxin domain-containing protein	TXN2	0	0	− 2.01
A0A287BDT8	Stearoyl-CoA desaturase	SCD	0	0	− 1.9
A0A4X1W2X5	Butyryl-CoA dehydrogenase	IVD	0	0	− 1.71
F1RPH0	Exostosin 1	EXT1	0	0	− 1.63
A0A480QDJ5	Hexosyltransferase	B3GALT5	0	0	− 1.61
A0A4X1TT31	Transducin Beta-Like Protein 1X	TBL1X	0	0	− 1.6
A0A5K1UCS8	Acyl-CoA-binding protein	DBI	0	− 3.57	− 1.47
A0A4X1V8G3	Cytochrome c oxidase subunit	COX6B	0	− 1.1	− 1.42
A0A287A8M1	NADH ubiquinone oxidoreductase 9 kDa subunit	NDUFV3	0	0	− 1.42
P00355	NAD(P)H-hydrate epimerase	NAXE	0	0	− 1.22
A0A287A1Y5	Aspartate aminotransferase, cytoplasmic	GOT1	0	− 3.22	0
F1SUE3	Malic enzyme	ME1	0	− 2.77	0
A0A4X1TEA2	Glutathione synthetase	GSS	0	− 2.49	0
A0A4X1TPV9	Aldo–keto reductase family 1 member A1	AKR1A1	0	− 2.18	0
A0A481CLJ7	S-formylglutathione hydrolase	ESD	0	− 2.11	0
A0A480X8T8	Adenylosuccinate lyase	ADSL	0	− 1.95	0
A0A287A808	S-(hydroxymethyl)glutathione dehydrogenase	ADH5	0	− 1.91	0
A0A0B8RVZ5	Leukotriene A(4) hydrolase	LTA4H	0	− 1.74	0
A0A287AA47	Triosephosphate isomerase	TPI1	0	− 1.7	0
A0A287AWS9	Transketolase	TKT	0	− 1.7	0
A0A287BQP2	Farnesyl pyrophosphate synthase isoform a	FDPS	0	− 1.66	0
A0A480TFA5	Thioredoxin-disulfide reductase	TXNRD1	0	− 1.66	0
A0A480UCJ4	Phosphoglycolate phosphatase	PGP	0	− 1.62	0
A0A480VNV2	Acyl-CoA Thioesterase 7	ACOT7	0	− 1.59	0
A0A4X1T9M8	Phosphoglycerate kinase	PGK1	0	− 1.56	0
A0A4X1TYP2	28S ribosomal protein S15, mitochondrial	MRPS15	0	− 1.49	0
A0A4X1TZ03	ML domain-containing protein	GM2A	0	− 1.47	0
A0A4X1U393	3-hydroxy-3-methylglutaryl coenzyme A synthase	HMGCS1	0	− 1.46	0
A0A4X1U9G6	UDP-glucose ceramide glucosyltransferase	UGCG	0	− 1.45	0
A0A4X1UTQ9	Isocitrate dehydrogenase [NADP]	IDH1	0	− 1.43	0
A0A4X1VLC0	Glyceraldehyde-3-phosphate dehydrogenase	GAPDH	0	− 1.43	0
A0A4X1VNK1	Mediator of RNA polymerase II transcription subunit 16	MED16	0	− 1.42	0
A0A4X1VYI7	Inorganic diphosphatase	PPA1	0	− 1.42	0
A0A4X1VYQ8	Adenine phosphoribosyltransferase	APRT	0	− 1.41	0
A0A4X1W580	Fructose-bisphosphate aldolase	ALDOC	0	− 1.4	0
A0A4X1WBQ4	1,2-dihydroxy-3-keto-5-methylthiopentene dioxygenase	ADI1	0	− 1.39	0
A0A5G2QAF0	BTB Domain And CNC Homolog 1	BACH1	0	0	1.27
A0A5G2QNU5	Cytidine deaminase	CDA	0	0	1.34
A0A5G2QPA8	Carnitine O-palmitoyltransferase 1A	CPT1A	0	0	1.48
A0A5G2R8S6	Fatty acyl-CoA reductase	FAR1	0	0	1.59
D2D0E3	Patatin Like Phospholipase Domain Containing 2	PNPLA2	0	0	1.62
F2Z5T7	Perilipin 3	PLIN3	0	0	1.71
Q5BLZ2	Glutamate-cysteine ligase modifier subunit	GCLM	0	0	2
U5HTD3	1-acylglycerol-3-phosphate O-acyltransferase	ABHD5	0	0	2.14
A0A1L1YNR3	Heme oxygenase 1	HMOX1	0	0	3.24
A0A287B0C7	Hydrolase_4 domain-containing protein	MGLL	1.25	0	1.43
A0A4X1T8J4	Epoxide hydrolase 1	EPHX1	2.81	0	0
G0Z3A1	Perilipin 2	PLIN2	5.09	0	5.74

### Common candidates between proteomics and RNA-seq analysis

**Figure 6:**
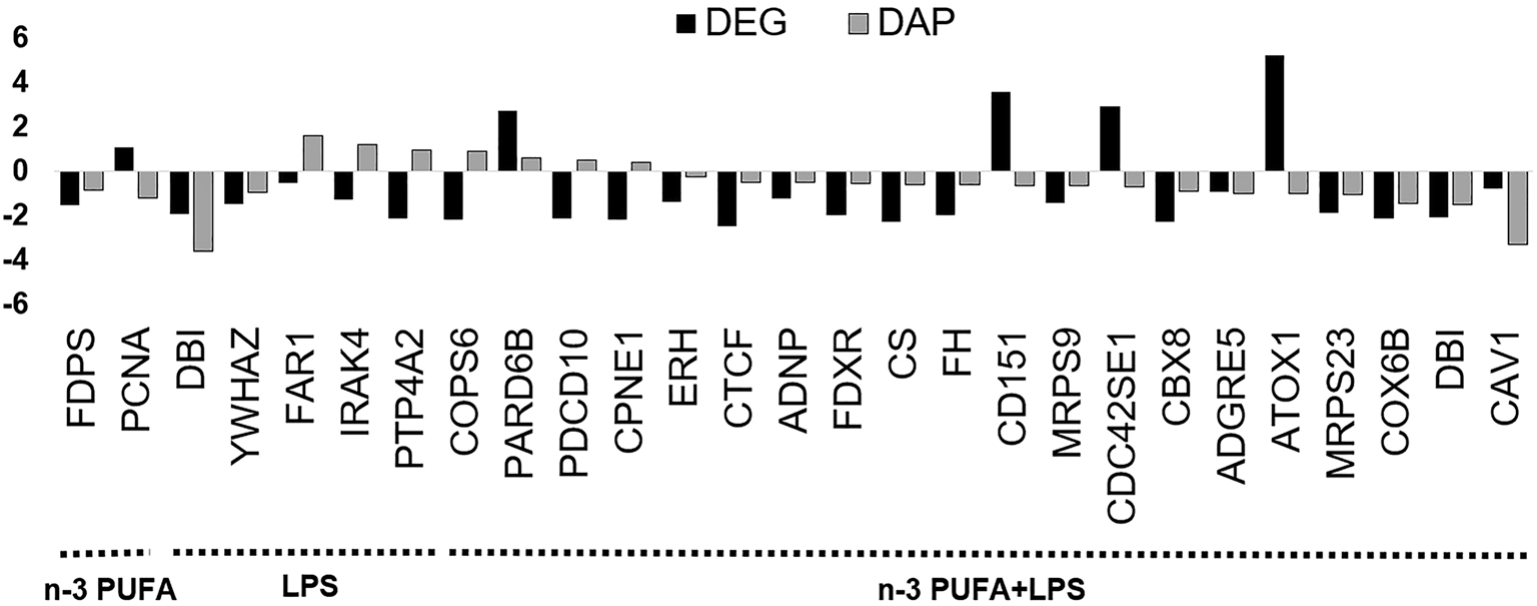
Candidates commonly identified between proteomics and RNA-seq analysis after applying log_2_FC ≥ 1.2 or ≤ − 1.2 and FDR ≤ 0.05 statistical filters. DEGs: Differentially expressed genes; DAPs: Differentially abundant proteins.

In total, 27 candidates were found in common between proteomics and RNA-seq analysis post filtering with log_2_FC ≥ 1.2 or ≤ − 1.2 and FDR ≤ 0.05 cut-offs (Fig. 6). Specifically, 23 common candidates were identified in the cells exposed to n-3 PUFA + LPS treatment, while only 2 were identified in n-3 PUFA and LPS treatments (Fig. 6). Between both omics analyses, 16 candidates were negatively regulated in parallel. Further, the mRNA levels of 5 candidates as PCNA (Proliferating Cell Nuclear Antigen), ATOX1 (Antioxidant 1 copper chaperone), CD151, CDC42SE1 (CDC42 small effector 1), and PARD6B (Par-6 family cell polarity regulator beta) were increased, while their protein abundance was decreased. On the contrary, protein levels of FAR1 (Fatty acyl-CoA reductase 1), IRAK4 (Interleukin 1 receptor associated kinase 4), PTP4A2 (Protein tyrosine phosphatase type IVA, member 2), COPS6 (COP9 signalosome subunit 6), PDCD10 (Programmed cell death 10), and CPNE1 (Copine 1) were increased compared to their mRNA levels that were decreased.

## Discussion

In this study, a proteomics approach integrated with RNA-seq technology was employed to comprehensively evaluate the molecular properties of n-3 PUFA in defending LPS-induced damage in porcine IPEC-J2 cells. Under different treatment conditions, changes in the abundance of proteins that primarily involved in central carbon metabolic process, such as the glycolysis/gluconeogenesis, oxidative phosphorylation (OXPHOS), and pentose phosphate pathway (PPP) were observed. Glycolysis is the process of converting glucose into pyruvate to produce ATP^20,21^. Glucose is the principal source of energy for most cellular biochemical processes. Conversely, gluconeogenesis is the reverse process, synthesizing glucose from pyruvate. Gluconeogenesis mainly occurs during high energy demands or under-starvations^22^. Glycolysis and gluconeogenesis pathways are closely and reciprocally regulated in the cytoplasm, particularly to support cellular energy requirements; therefore, they share several catalytic enzymes as observed in the present study^22^. Specifically, LPS reduced the abundance of key regulatory enzymes (ALDOC, GADPH, LDHA, PGK1, ADH5, and TPI1) that control the glycolysis/gluconeogenesis pathway^21,23^. The enzyme ALDOC participates in the initial steps of glycolysis converting fructose-1,6-biphosphate into dihydroxyacetone phosphate (DHAP). Then, TPI1 converts DHAP into glyceraldehyde 3-phosphate (G3P). Subsequently, GAPDH catalyzes the conversion of G3P into 1,3-biphosphoglycerate which is converted to 3-phosphoglycerate by PGK1^21,23^. At the end of glycolysis, pyruvate and ATPs are generated. Apart from pyruvate, gluconeogenesis also utilizes amino acids for glucose synthesis. Apparently, the abundance of proteins involved in amino acid biosynthesis (ALDOC, GAPDH, GOT1, IDH1, PGK1, TKT, and TPI1) were also inhibited by LPS treatment. This impact could be associated with gluconeogenesis dysregulation observed in the LPS-stimulated cells. When cells were treated with n-3 PUFA before the LPS stimulation, the disruption of glycolysis/gluconeogenesis and amino acid synthesizing enzymes were significantly reduced.

During anaerobic conditions, LDH converts the pyruvate into lactate to produce ATP in the cytoplasm. But under aerobic conditions, pyruvate enters the mitochondria to fuel OXPHOS for ATP production^20,21^. In the present study, LPS reduced the abundance of enzymes involved in both anaerobic (LDHA) and aerobic glycolysis leading to OXPHOS (COX6B, DBI, and ND1). Interestingly, n-3 PUFA supplementation also moderately decreased the abundance of enzymes corresponding to OXPHOS (COX6B, ND1, and NDUFV3) in infected cells. In cells not stimulated with LPS, n-3 PUFA did not regulate these enzymes. Generally, OXPHOS produces 36 ATP molecules, whereas glycolysis produces only 2. Therefore, 70% of the cellular energy requirement is supplied by mitochondrial OXPHOS^20^. Although glycolysis generates less energy, it is more rapid than OXPHOS^20,24^. For this reason, cancer cells switch their energy metabolism from OXPHOS to glycolysis, to cope with their energy requirement for rapid cell division^25,26^. This property of cancer cells and some immune cells in switching from OXPHOS to glycolysis is denoted as Warburg effect^26^. As n-3 PUFA shifted the energy metabolism only in infected cells, possibly it executed Warburg-like effect to protect the cells from LPS damage.

Cells generally store or hideaway excessive circulating triacylglycerol and cholesterol esters into lipid droplets for maintaining lipid homeostasis^27,28^. During high energy demands or under starvation, cells utilize the fat stored in lipid droplets as energy source^27,28^. Perilipins (PLIN2 and/or PLIN3), whose abundance was significantly increased by n-3 PUFA in our study, are involved in the formation of intracellular lipid droplets^27,28^. In LPS-stimulated cells, n-3 PUFA increased the abundance of specific hydrolases (ABDH2, ABDH5, and PNPLA2) that breaks the lipid droplets into free fatty acids and glycerols^28,29^. Additionally, n-3 PUFA reduced the abundance of 4 essential enzymes (FADS1, FADS2, FADS4, and FADS5) from the family of FADS in infected cells. These enzymes play an important role in the synthesis of unsaturated fatty acids^30–32^. In cells not exposed to LPS, n-3 PUFA enhanced the abundance of only 2 enzymes, PLIN2 and ABDH2. Earlier, the PNPLA family of hydrolases was reported to act as a co-activator of ABDH5 for interacting with the perilipins during hydrolysis^28,29^ These observations suggest that n-3 PUFA enables lipid storage by controlling perilipin abundance under normal cellular conditions. Conversely, after LPS stimulation, n-3 PUFA reduced the fatty acid synthesis and promoted the breakdown of lipid droplets by enhancing hydrolase abundance. Subsequently, fatty acids and glycerol released from lipid droplets could have been utilized by IPEC-J2 cells to counterbalance the LPS-disrupted glycolysis.

The PPP metabolic pathway branches out in the very first step of glycolysis, starting from glucose-6-phosphate (G6P), generating two metabolic molecules as the ribose 5-phosphate (R5P) and nicotinamide adenine dinucleotide phosphate (NADPH) that destinated for other cellular functions^33^. Specifically, R5P acts as a precursor for nucleic acid synthesis, whereas NADH assists in fatty acid, nucleotide, and non-essential amino acid synthesis^34^. More importantly, NADPH regulates the glutathione (GSH)-mediated antioxidant defense system^35^. In our study, LPS reduced the abundance of specific proteins involved in PPP (ADSL, ALDOC, APRT, GAPDH, ME1, PGK1, TKT, and TPI1) and GSH defense system (GSS). Intriguingly, n-3 PUFA treatment prior to the LPS stimulation significantly prevented the inhibition of these proteins. Moreover, n-3 PUFA enhanced the abundance of GCLM, the first rate-limiting enzyme in GSH synthesis^36^. GCLM mainly synthesizes glutathione from L-cysteine and L-glutamate^36^. Possibly, n-3 PUFA counterbalanced the LPS-mediated disruption of GSH synthesis by increasing the abundance of GCLM. However, interestingly, n-3 PUFA also reduced the abundance of OPLAH enzyme that synthesises L-glutamate by cleaving oxo-L-proline at the expense of ATP^37^. These observations imply that for energy conservation during stress conditions, such as in LPS stimulation, n-3 PUFA enabled the cells to convert available L-glutamate into GSH via GCLM instead of synthesising a new precursor for GSH synthesis via OPLAH.

In the comparative analysis between proteomics and RNA-seq data, 27 genes were highlighted in two analyses. Sixteen of these genes (FDPS, DBI, YWHAZ, PARD6B, ERH, CTCF, ADNP, FDXR, CS, FH, MRPS9, MRPS23, CBX8, ADGRE5, COX6B, and CAV1) exhibited a positive correlation between their mRNA levels and protein abundance, considerably validating the present observations. Five proteins (PCNA, ATOX1, CD151, PARD6B, and CDC42SE1) exhibited higher mRNA levels than their protein abundance, suggesting a higher transcription rate in these candidates. Of note, PARD6B is an important membrane protein localized at the tight junctions of intestinal epithelium^38^. While CDC42 is one of the three members of Rho-family GTPases, that regulates the formation of the apical cell–cell junction and apical-basal polarization by interacting with PARD6B^38,39^. On the contrary, 6 genes (FAR1, IRAK4, PTP4A2, COPS6, PDCD10, and CPNE1) exhibited higher protein abundance than their mRNA levels. This behavior may imply that multiple genes have encoded the same protein, lower mRNA stability in these candidates, or post-translational mechanisms are prolonging the protein half-life^40^ compared to mRNA expression. Also, cells could have been in different stages of gene expression when mRNA/proteins were isolated for each omics analysis.

Finally, many proteins such as the IRAK3/4, MyD88 (Myeloid differentiation primary response 88), IKBKB (Inhibitor of nuclear factor kappa-B kinase subunit beta), IKBKG (Inhibitor of nuclear factor kappa-B kinase regulatory subunit gamma), and NFRKB (Nuclear factor related to kappa-B binding protein) were also identified. These proteins are implicated in LPS-mediated Toll-like receptor (TLR) inflammatory signaling^41^. However, except IRAK4, the abundance of other inflammatory proteins was not significantly different from the control, although they were detected. A possible reason could be that the cells had already crossed the inflammatory stage at the point of protein isolation. In support of this observation, earlier infected cells were reported to acutely enhance the metabolism, particularly glycolysis (Warburg-like effect) to meet the energy requirement of inflammatory process^24,26,42^. Infected cells also enhance fatty acid synthesis to produce lipid-based signaling mediators such as cytokines^43–45^. At the later stages of inflammation, infected cells were reported to switch back to glycolysis from OXPHOS (reverse of Warburg) in facilitating cellular homeostasis^24,26^. In the present study, detection of inflammatory proteins below the significance level and dysregulation of the entire metabolism, including fatty acid synthesis, implies that LPS has stimulated cell starvation. On the other hand, n-3 PUFA pre-treatment was able to recover the cell starvation by restitution of glycolysis/gluconeogenesis in these cells. Similar to our proteomic results, in another in vitro study *Salmonella enterica* serovar Typhimurium was shown to abrogate the glycolysis and insulin signaling to impair the macrophage defence system^46^. Likewise, liver and faecal metabolomic profiles of *S. Typhimurium*-infected mice exhibited a profound disruption in the entire branch of eicosanoid and steroid metabolic pathways^47^. Notably, LPS used in the present study was also extracted from *S. Typhimurium*. This aspect could partly explain our current observations concerning the metabolic dysregulation of IPEC-J2 cells, post-challenge with LPS.

**Figure 7:**
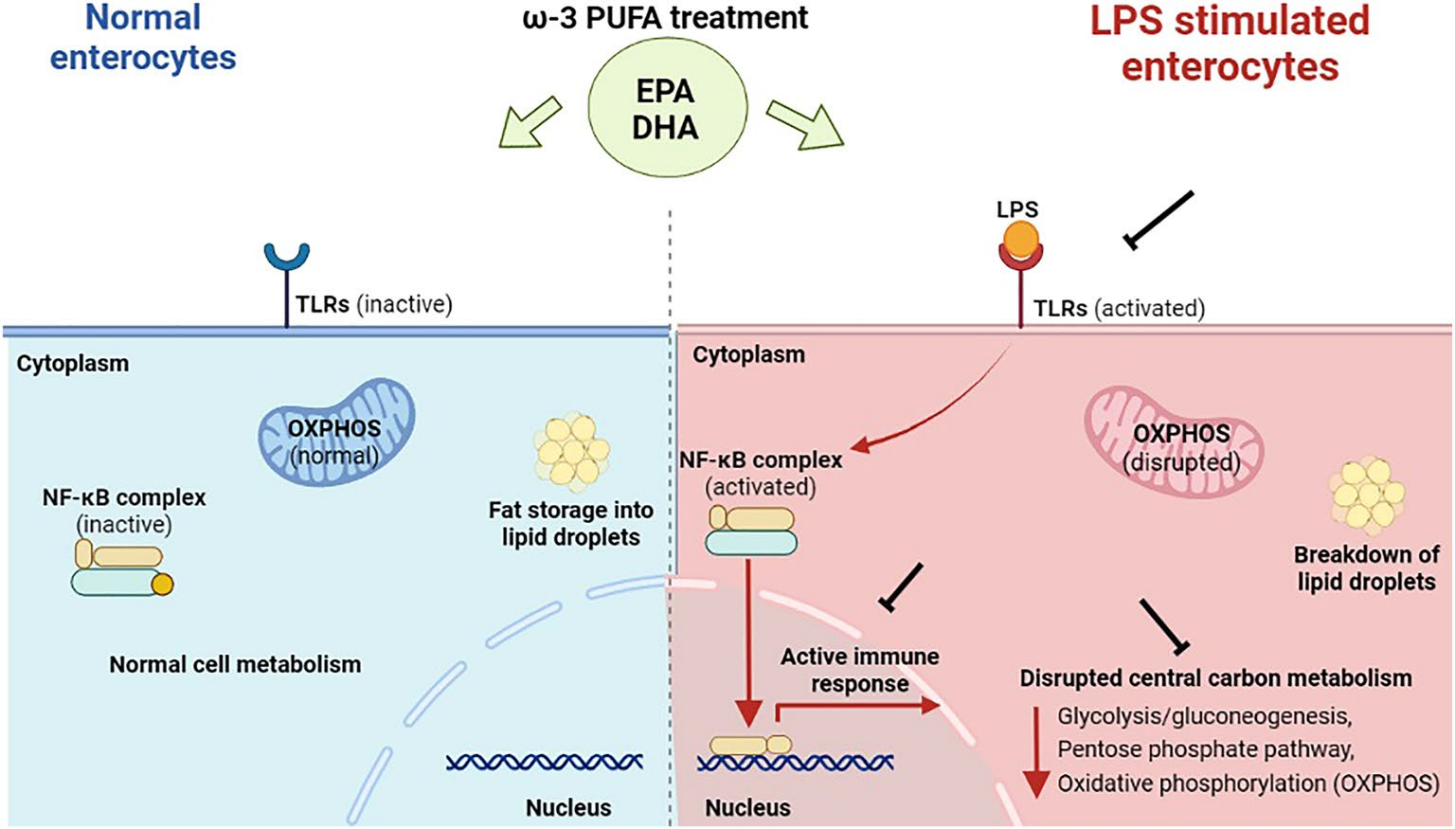
Schematic representation highlighting the n-3 PUFA activity in porcine enterocytes, in the presence and absence of LPS stimulation. Red line denotes the pathways activated by LPS, while black denotes the pathways secured by n-3 PUFA. Figure created with BioRender.com.

In conclusion, this study has attempted to comprehensively map the biological pathways regulated by n-3 PUFA in the porcine enterocyte model, particularly under LPS stress conditions. Accordingly, challenge with LPS was shown to downregulate the enterocyte’s central carbon metabolic process. Prior treatment with n-3 PUFA contrasted the LPS-stimulated metabolic dysregulation. Based on differential proteomic analysis, it is speculated that n-3 PUFA could have stimulated hydrolysis of fat stored in lipid droplets to generate energy for counterbalancing the metabolic damage in infected cells. On the other hand, n-3 PUFA enhanced fatty acid storage into lipid droplets in unstimulated cells for the maintenance of lipid homeostasis. The major findings of this study are highlighted in Fig. 7. Furthermore, as omics-based investigations do not support the analysis of both genes and proteins from the same cell samples, some candidates exhibited poor correlation between their expression profiles in the comparative analysis. This is one of the limitations of present study that could affect the interpretation of their role in cell metabolic pathways. Overall, to the best of our knowledge, this is the first study to reveal the distinct behavior of n-3 PUFA between normal and LPS-stimulated enterocytes using shotgun proteomics analysis, integrated with RNA-seq validation. The data generated from the current study may provide nutritional biomarkers for monitoring gut health, immunity, and metabolic status corresponding to n-3 PUFA-based diet in human and animals. Further studies using other omics technologies such as the metabolomics, could be utilized to substantiate the present outcomes on the metabolic status of enterocytes upon n-3 PUFA treatment.

## Materials and methods

### Cell culture and treatment conditions

IPEC-J2 cell line is a model of small intestinal absorptive cell type, isolated from the jejunal epithelium of unsuckled piglets (DSMZ, Braunschweig, Germany). IPEC-J2 cells were cultured in a complete medium consisting of a 1:1 mixture of Dulbecco’s modified Eagle’s medium with stable L-Glutamate and Ham’s F-12 nutrient mixture (DMEM/F12) (Immunological sciences, Rome, Italy), supplemented with 15 mM HEPES (Sigma-Aldrich, Milan, Italy), 5% FBS (Immunological Sciences, Rome, Italy) and 1% penicillin–streptomycin (Euroclone, Milano, Italy). Cell cultures were maintained in a humidified atmosphere at 37 °C under 5% CO_2_. EPA and DHA (Sigma-Aldrich, Milan, Italy) were dissolved in ethanol to produce a 100 mM stock solution. For proteomics analysis, cells were seeded at a density of 1.5 × 10^6^ in 75 cm^2^ culture flasks to obtain a monolayer with 80% confluency after 24 h. Cells were then treated with or without n-3 PUFA (DHA:EPA, 1:2 ratio, 10 µM) that reconstituted in the DMEM media with 0.05% FBS for 24 h. Subsequently, cells were challenged with LPS (10 µg/mL) (Sigma-Aldrich, Milan, Italy) in DMEM media for 24 h. Control cells contained 0.1% ethanol, according to maximum volume in the treatments. Each treatment group was composed of four biological replicates. After the treatments, cells were rinsed thrice with 1 × PBS and stored at -80 °C until used.

### Cell lysis and protein extraction

Cells in culture flask were harvested in lysis buffer (2% SDS, 0.1 M Tris–HCl pH 6.8, 0.1 M dithiothreitol (DTT), 1 × protease inhibitor) and incubated at 95 °C for 15 min. Subsequently, cell samples were sonicated for 10 min and then centrifuged at 15,000 × g for the same time. Protein concentration was estimated using the Pierce 660 nm Protein Assay Kit with a pre-diluted BSA standard (Thermo Scientific), following the manufacturer’s instructions.

### Protein digestion

Protein samples were subjected to reduction, alkylation, and trypsin digestion following the filter-aided sample preparation (FASP) protocol as described earlier^48,49^. In brief, samples were diluted in the Tris-Urea buffer (8 M urea, 0.1 M Tris–HCl, pH 8.8) and loaded onto Amicon Ultra-0.5 centrifugal filter units (3 kDa cut-off membrane, Millipore, Billerica, MA, USA), followed by centrifugation at 13,000 × g for 15 min. All buffer exchanges were performed at identical centrifugation conditions. The concentrates obtained were diluted in the same buffer and centrifuged again. Proteins in the filter were reduced with 10 mM DTT in the Tris-Urea buffer for 30 min and then alkylated with 50 mM iodoacetamide (IAA) in the same buffer for 20 min. After six washes (three times in Tris-Urea buffer and three times in 50 mM Ammonium bicarbonate), trypsin solution was dispensed onto samples in the filter (1:100 enzyme-to-protein ratio) and incubated overnight at 37 °C. After trypsin digestion, the peptides were collected by centrifugation and washed with an elution solution (20% acetonitrile and 1% formic acid). Finally, the peptide mixtures were dried and reconstituted in 0.2% formic acid to a final concentration of 2 µg/µL. The concentration of the peptide mixtures was estimated by measuring the absorbance at 280 nm in a NanoDrop 2000 spectrophotometer (Thermo Scientific, San Jose, CA, USA) with MassPREP *E. coli* Digest as the standard (Waters, Milford, MA, USA).

### LC–MS/MS analysis

Peptide analysis was carried out using a Q-Exactive mass spectrometer (Thermo Scientific) interfaced with an UltiMate 3000 RSLCnano LC system (Thermo Scientific) as described earlier^48,49^. In brief, all peptide mixtures (4 μg per run) were concentrated and desalted on a trapping pre-column (Acclaim PepMap C18, 75 μm × 2 cm nanoViper, 3 μm, 100 Å, Thermo Scientific), using 0.2% formic acid at a flow rate of 5 μL/min. Subsequently, peptides were separated at 35 °C using a C18 column (Acclaim PepMap RSLC C18, 75 μm × 50 cm nanoViper, 2 μm, 100 Å, Thermo Scientific) at a flow rate of 250 nL/min with a 245 min gradient from 2 to 37.5% eluent B (0.2% formic acid in 20% acetonitrile) in eluent A (0.1% formic acid). MS data was acquired using a data-dependent Top12 method by dynamically choosing the most abundant precursor ions from the survey scan under the direct control of Xcalibur software (version 3. 1.66.10 SP2), where a full-scan spectrum (from 300 to 2,000 m/z) was followed by tandem mass spectra (MS/MS). The instrument was operated in positive mode with a spray voltage of 1.8 kV and a capillary temperature of 275 °C. Survey and MS/MS scans were performed in the Orbitrap with a resolution of 70,000 and 17,500 at 200 m/z, respectively. The automatic gain control was set to 1,000,000 ions. For accurate mass measurements, the lock mass option was enabled on a protonated polydimethylcyclosiloxane background ion as an internal recalibration. The dynamic exclusion was set to 30 s. Fragmentation occurred by Higher Energy Collisional Dissociation (HCD) at 25 eV, using nitrogen as the collision gas.

### Data processing

Proteins were identified using the Proteome Discoverer (version 2.4, Thermo Scientific) with Sequest-HT as search engine. Raw files were processed with the following parameters: Database UniprotKB: taxonomy *Sus scrofa* (Taxon identifier: 9823, 120, 707 sequences, release_2021_03); Enzyme: trypsin, with two missed cleavages allowed; Precursor mass tolerance: 10 ppm; Fragment mass tolerance: 0.02 Da; Charge states: + 2, + 3, and + 4; Static modification: cysteine carbamidomethylation; Dynamic modification: methionine oxidation and acetylation (Acetyl), loss of Methionine (Met-loss) and loss of Methionine-loss + Acetylation (Met-loss + Acetyl) on N-Terminal. Protein significance and peptide validation (false discovery rate: FDR < 1%) were performed with the percolator algorithm^50,51^. Peptide and protein grouping were allowed according to the Proteome Discoverer algorithm by applying the strict maximum parsimony principle.

### Detection of differentially abundant proteins

Differential analysis was performed by comparing each experimental group against the control. A consensus workflow was set on the Proteome Discoverer 2.4 to determine the precursor ions by label-free quantification based on the spectral counting (SpC) approach as described earlier^52,53^. Precursor ion abundance was calculated using intensity as an abundance parameter, which was normalized for determining the significance of the abundance ratio among proteins identified in two different experimental groups. Precursor ion quantification was performed only if the ions were detected in at least 50% of the samples in an experimental group. The fold change ratio of protein abundance was calculated by pair-wise comparison in which the maximum ratio was set to 100. Statistical significance of differences in protein abundance was assessed by t-test with FDR correction, following the Benjamini–Hochberg method. Only the proteins with log twofold change (log_2_FC) value ≥ 1.2 or ≤ − 1.2 and adjusted *p*-value ≤ 0.05 were considered statistically significant.

### Bioinformatics data analysis

Protein accession IDs were converted to gene names before the data analysis using the UniProt database. Some unidentified proteins were then compared against other mammals such as *Homo sapiens* for matching proteins using the Basic Local Alignment Search Tool (BLAST) (https://www.uniprot.org/blast) search option in UniProt. Only the proteins that matched ≥ 80% with 0.01 E-Threshold were included for further analysis. Biological processes in which the DAPs participate were classified by Gene Ontology (GO) annotation analysis using the ShinyGO (version 0.76, http://bioinformatics.sdstate.edu/go/) web application. GO analysis was performed by setting the inbuilt FDR option to < 0.05. Pathway enrichment analysis was performed using the open-source Reactome database (https://reactome.org/). The pathways that exhibited *p*-value ≤ 0.05 was considered statistically significant. For visualizing the distribution of DAPs among the treatment groups, bar graphs and volcano plots were generated using Microsoft Excel, and the Venn diagrams using molbiotool application (https://molbiotools.com/). Protein–protein interaction (PPI) network was built using the STRING database^54^ with a minimum required interaction score of 0.7 (high confidence) and *p-*value ≤ 0.05. STRING provides information by retrieving data from other pathway databases such as Kyoto Encyclopedia of Genes and Genomes (KEGG), Reactome, Gene Ontology Resources, and Pfam.

### Comparative analysis with RNA-seq data for verification of common candidates

For verification, proteomics results were compared against RNA-seq data obtained under the same experimental conditions. Specifically, candidates between both analyses were compared only if either of them exhibited log_2_FC ≥ 1.2 or ≤ − 1.2 and adjusted *p*-value ≤ 0.05. For RNA-seq, samples were processed and analysed as reported earlier in detail^55,56^. In brief, after cellular treatments, mRNA was isolated using RNeasy Mini Kit (Qiagen, Germany) according to the manufacturer’s instructions. RNA concentration was measured using a Nanodrop (Thermo scientific) and RNA integrity by capillary electrophoresis (Fragment Analyzer, Advanced Analytical Technologies, USA). Subsequently, RNA samples were retro transcribed using mRNA-Seq Library Prep Kit (Lexogen, Austria) as per the manufacturer’s instructions. The double stranded DNA libraries were subjected to purification, amplification and then sequencing on Illumina NextSeq platform exactly as described earlier^55,56^. Fastq files were processed and aligned to the reference genome (*Sus scrofa*) using STAR aligner (https://github.com/alexdobin/STAR/releases/tag/2.5.2b). The low read counts (< 3 count per million) were removed from further analysis. The differentially expressed genes (DEGs) were sorted using edgeR in R package (https://bioconductor.org/packages/ release/bioc/html/edgeR.html) by applying log_2_FC ≥ 1.2 or ≤ − 1.2 and FDR ≤ 0.05 cut-offs.

### Supplementary Information

Supplementary Information.

## Data Availability

The datasets generated and/or analysed during the current study were deposited to the public repository, PRIDE (https://www.ebi.ac.uk/pride/) with the identifier PXD036456.

## Abbreviations

ATP: Adenosine triphosphate
DAPs: Differentially abundant proteins
DHA: Docosahexaenoic acid
EPA: Eicosapentaenoic acid
FADS: Fatty acid desaturase
FASP: Filter-aided sample preparation
GO: Gene Ontology
GSH: Glutathione
KEGG: Kyoto encyclopedia of genes and genomes
LDH: Lactate dehydrogenase
LPS: Lipopolysaccharides
n-3 PUFA: Omega-3 polyunsaturated fatty acid(s)
OXPHOS: Oxidative phosphorylation
PLIN: Perilipin
PPI: Protein–protein interaction
PPP: Pentose phosphate pathway
RNA-seq: RNA-sequencing
